# Zero-temperature quantum annealing bottlenecks in the spin-glass phase

**DOI:** 10.1038/ncomms12370

**Published:** 2016-08-05

**Authors:** Sergey Knysh

**Affiliations:** 1 QuAIL, NASA Ames Research Center, Moffett Field, California 94035, USA; 2 SGT Inc., 7701 Greenbelt Road, Suite 400, Greenbelt, Maryland 20770, USA

## Abstract

A promising approach to solving hard binary optimization problems is quantum adiabatic annealing in a transverse magnetic field. An instantaneous ground state—initially a symmetric superposition of all possible assignments of *N* qubits—is closely tracked as it becomes more and more localized near the global minimum of the classical energy. Regions where the energy gap to excited states is small (for instance at the phase transition) are the algorithm's bottlenecks. Here I show how for large problems the complexity becomes dominated by *O*(log *N*) bottlenecks inside the spin-glass phase, where the gap scales as a stretched exponential. For smaller *N*, only the gap at the critical point is relevant, where it scales polynomially, as long as the phase transition is second order. This phenomenon is demonstrated rigorously for the two-pattern Gaussian Hopfield model. Qualitative comparison with the Sherrington-Kirkpatrick model leads to similar conclusions.

## Abstract (web summary)

Quantum annealing can solve hard optimization problems, but it is limited by computational bottlenecks. Here, the author obtains the scaling of spin-glass bottlenecks with the problem size and explains a crossover to exponential complexity for large sizes.

## Graphical abstract

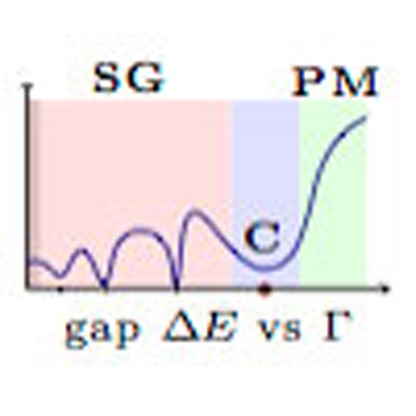

Quantum algorithms offer hope for tackling computer science problems that are intractable for classical computers^1^. However, exponential speed-ups seen in, for example, number factoring^2^, have not materialized for more difficult non-deterministic polynomial time (NP)-complete problems^3^. Those problems are targeted by the quantum adiabatic annealing algorithm (QA)^4^,^5^,^6^. Any NP-hard problem can be recast as quadratic binary optimization. QA solves it by implementing a quantum Hamiltonian, written with the aid of Pauli matrices as


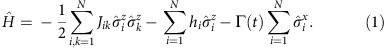


Here the first two terms, diagonal in *z*-basis, encode the objective function. The last term represents the magnetic field in the transverse direction, which is decreased from Γ(0)≫1 to Γ(*T*_ann_)=0. The time *T*_ann_ needed by the algorithm is determined by a condition that the annealing rate is sufficiently low to inhibit non-adiabatic transitions:


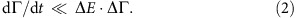


These are most likely near points where the instantaneous gap to excited states Δ*E* attains a minimum as a function of Γ; further, ΔΓ is defined as the width of the region where the gap remains comparable to its minimum value.

QA offers no worst-case guarantees on time complexity^7^, but initial assessments of typical case complexity were optimistic. Both experimental^8^ and theoretical^9^ evidence hinted at performance improvement over simulated annealing for finite-dimensional glasses; however, some empirical evidence in support of the theory has recently been called into question^10^. Early exact diagonalization studies also observed polynomially small gaps in the constraint satisfaction problem (CSP) on random hypergraphs^11^, but that finding had been challenged by quantum Monte Carlo studies involving larger sizes^12^. Benchmarking of a hardware implementation of QA, courtesy of D-Wave Systems, shows no improvement in the scaling of the performance^13^,^14^. Whether that might be attributable to a finite temperature at which the device operates or its intrinsic noise is unclear at present^15^,^16^,^17^.

Statistical physics offers some intuitive guidance: Small gaps develop at the quantum phase transition point and become exponentially small when the transition is first-order^18^,^19^,^20^,^21^ or when different parts of the system become critical at different times for strong-disorder continuous phase transitions^22^. The most promising candidates for QA are thus problems with bona fide second-order phase transitions, where the disorder is irrelevant at the quantum critical point (QCP).

The scaling analysis described here suggests a polynomially small gap at the critical point of the archetypal spin glass: the Sherrington-Kirkpatrick (SK) model^23^,^24^,^25^. It has been pointed out^9^,^26^ that QA may still be doomed by the bottlenecks in the spin-glass phase. Exponentially small gaps away from the critical point have been observed in simulations^27^, but adequate theoretical description of this phenomenon has proven challenging. A perturbative argument in support of this qualitative picture has been considered in ref. 26. However, the results were not borne out by more accurate analysis that took into account the extreme value statistics of energy levels^28^.

The present manuscript sheds light on the mechanism of tunnelling bottlenecks in the spin-glass phase. Using exact, non-perturbative, methods, this is illustrated for a simple model, but the main findings are expected to be valid for quantum annealing of more realistic spin glasses. During annealing, the system must undergo a cascade of tunnellings at some specific values of Γ_1_,Γ_2_,… in an approximate geometric progression. For a finite system size, these bottlenecks are few, *O* (log *N*), and may not even appear until *N* is sufficiently large, highlighting the challenge of interpreting the results of numerical studies. Bottlenecks also become increasingly easier as Γ→0, counter to expectations that tunnellings are inhibited as the model becomes more classical. A related finding is that the time complexity of QA is exponential only in some fractional power of problem size: a mild improvement over more pessimistic estimates^26^.

## Results

### Summary

The spin-glass phase, which is entered below some critical value of the transverse field Γ_c_, is characterized by a large number of valleys. Often, this transition is abrupt, driven by competition between an extended state and a valley (localized state) with the lowest energy, as occurs in the random energy model^19^,^20^. The exponentially small overlap between the two states then determines the gap at the phase transition. However, even if new valleys develop in a continuous manner as Γ decreases, small changes in the transverse field may result in a chaotic reordering of associated energy levels, leading to Landau–Zener avoided crossings and attendant exponentially small gaps.

Nonetheless, attempts to make this intuition exact are fraught with potential pitfalls. For increasing Γ, two randomly chosen valleys are equally likely to come either closer together or further apart in energy. In the case of the former—and further, if the sensitivity of energy levels to changes in the transverse field is so large that the levels ‘collide' before either valley disappears—avoided crossing will occur. This may not be necessarily the case when one considers ‘collisions' with the ground state, which are of particular concern to QA. The ground state corresponds to a valley with the lowest energy; this and other low-lying valleys obey fundamentally different statistics of the extremes.

A case in point is the analysis of ref. 26, which develops perturbation theory in Γ. The classical limit (Γ=0) is used as a starting point; how that analysis might be extended to Γ>0 has also been discussed^29^. A type of CSP has been considered: classical energy levels are discrete non-negative integers (number of violated constraints) but have exponential degeneracy. ‘Zeeman splitting' for Γ>0 scales as 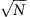, which, for large problems, may be sufficient to overcome the *O*(1) classical gap and cause avoided crossings of levels associated with different classical energies. Yet this trend disappears if only levels with the smallest energies (after splitting) are considered; these are relevant for avoided crossings with the ground state. This about-face is not immediately apparent, only coming into play for *N*≳100, when the exponential degeneracy of the classical ground state sets in. It has, however, been confirmed with analytical argument and numerics^28^. Consequently, arguments based on perturbation theory cannot be used to establish the phenomenon.

**Figure 1:**
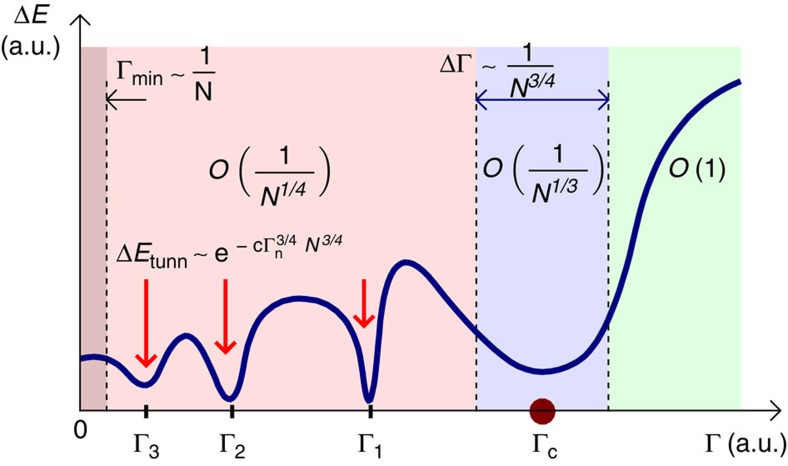
Scaling of the gap in various regions. Sketch of the behaviour of the gap in a Hopfield model with the Gaussian distribution of disorder variables (units are arbitrary): in the paramagnetic phase (Γ>Γ_c_), in the glassy phase (Γ<Γ_c_) and in the critical region (Γ≈Γ_c_). Scaling of the typical gap in these regions is indicated in bold letters using big-O notation. The area Γ<Γ_min_∼1/*N* is where the discrete nature of the energy landscape becomes manifest: the ground state becomes nearly completely localized. The glassy phase also contains log *N* isolated bottlenecks at Γ_1_, Γ_2_, Γ_3_, and so on. (indicated with red arrows), where the gaps scale as a stretched exponential. a.u., arbitrary units.

This manuscript offers a fresh perspective, illustrated by studying quantum annealing of the Hopfield model. Mean-field analysis correctly describes thermodynamics if the number of random ‘patterns' is small. The method is further extended to extract information about exact quantum energy levels. Importantly, the classical energy landscape is made much more complex by insisting that the distribution of disorder is Gaussian. Figure 1 sketches a ‘phase diagram' obtained for this model. For decreasing Γ the gap changes as follows: (1) it is finite (does not scale with *N*) in the paramagnetic phase, Γ>Γ_c_; (2) scales as 1/*N*^1/3^ in the narrow region of width 1/*N*^2/3^ around Γ=Γ_c_; (3) increases slightly, with typical values scaling as 1/*N*^1/4^ for Γ<Γ_c_. In addition, there are avoided crossings at isolated points Γ_1_,Γ_2_,….

The first non-trivial example requires two Gaussian patterns. In this case the ‘energy landscape' is effectively one-dimensional, which greatly simplifies the analysis. The most important element of this analysis is accounting for the extreme value statistics associated with the valleys (local minima) having the lowest energies. To this end, the distribution of energies of the classical landscape must be conditioned so that they are never below the energy of the global minimum. This becomes feasible when reformulated as a continuous random process, in the limit *N*→∞.

This particular model is not as interesting from the computer science perspective, not least because it affords an efficient classical algorithm. It is sufficiently simple so that a complete quantitative analysis presented later on in the manuscript has been possible. Yet, the model captures the essential properties of the spin glass: its qualitative features directly apply to much more general models, including Sherrington-Kirkpatrick. The most important feature of the classical energy landscape is that it exhibits fractal properties, which both ensures that hard bottlenecks are present in the spin-glass phase and also governs their distribution. The role of the transverse field is to approximately coarse-grain it on scales determined by Γ, eliminating small barriers; thus the number of valleys decreases as Γ grows. A specific random process, corresponding to the energy landscape of the ‘infinite'-size instance, will contain every possible realization of itself at some ‘length scale'. Some realizations will contain high barriers that cannot be easily overcome; these will lead to tunnelling bottlenecks.

This intuition can be used to immediately establish the scaling of the number of tunnelling bottlenecks. Since the model contains no inherent length scale in the limit *N*→∞, it can be argued that the expected number of tunnelling bottlenecks in a finite interval [Γ_1_;Γ_2_] should be a function of the ratio Γ_2_/Γ_1_. The logarithm is the only function that respects additivity, that is, 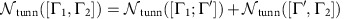. To obtain the total number of bottlenecks, one considers the interval [Γ_min_;Γ_c_]. Here Γ_c_∼1 is the boundary of the spin-glass phase. The lower cutoff, Γ_min_, corresponds to the lowest energy scale of the classical model, which scales as an inverse power of system size, for example, as 1/*N* for the Gaussian Hopfield model. In a sense, tunnelling bottlenecks are connected to the Γ=0 ‘fixed point' (note that the classical gap vanishes asymptotically). To summarize, the number of tunnelling bottlenecks will grow as


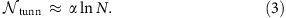


Locations (in Γ) will depend on specific disorder realization, but self-similarity ensures that the successive ratios Γ_*n*_/Γ_*n*+1_ converge to a universal distribution.

This logarithmic rise is far weaker than a power law seen in some phenomenological models of temperature chaos^30^ and, as has been argued above, likely to be a universal feature. The prefactor is model-dependent; its numerical value can be used to estimate the minimum problem size for which the mechanism becomes relevant, via 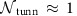. A value of *α*≈0.15 is obtained for the problem at hand, so that additional bottlenecks become an issue for *N*≳1,000. Prior numerical studies similarly required large sizes before the exponentially small minimum gap was observed^27^, and so far there has been no evidence of two or more exponentially small gaps coexisting. The slow, logarithmic increase of the expected number of bottlenecks is the most likely culprit.

A notable feature of these results is that tunnellings become progressively ‘easier' as Γ→0 despite the fact that the model becomes more classical. Tunnelling gaps increase as


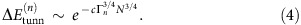


Notice that they cease to be exponentially small for Γ≲Γ_min_; at that point the ground state is already localized near the correct global minimum. The power law exponent for this stretched exponential is model-dependent, related to the scaling of barrier heights. These scale as *N*^1/2^ for the Gaussian Hopfield model, which, together with *O*(*N*) scaling of the effective mass, gives rise to the *N*^3/4^ term in the exponent.

The finding that the gaps increase for smaller Γ deserves explanation. Typically, valleys with similar energies differ by up to *N*/2 spin flips. This changes once lowest energies are considered: All spin configurations with energies less than 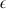 above the global minimum are contained in a neighbourhood of radius 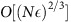, using Hamming distance as a metric. The problem is not rendered easy by the mere fact that the global minimum is so pronounced (although theoretical analysis inspired an efficient classical algorithm for the *p*=2 Hopfield network, briefly described later on). It does imply, however, that the ground state wavefunction does not jump chaotically: Every subsequent tunnelling involves shorter distances, with *O*(Γ*N*) spin flips, and achieves progressively better approximation to the true global minimum. Absent such a trend, annealing would be most difficult towards the end of the algorithm, when Γ∼1/*N*, and the minimum gap would exhibit less favourable exponential scaling^26^.

In what follows, the model and its solution are described in greater detail. First, finite-size scaling of the ‘easy' QCP bottleneck is linked to the thermodynamics of the phase transition. The next part goes beyond thermodynamics, considering small corrections to the extensive contribution to the free energy. The entire low-energy spectrum, which depends on disorder realization, is mapped onto that of a simple quantum mechanical particle in a random potential. Finally, extreme value statistics is applied to investigate the properties of that random potential near its global minimum by mapping it to a Langevin process. This yields the distribution of hard bottlenecks in a universal regime (Γ<<1).

### Quantum Hopfield network

Consider a model with rank-*p* matrix of interactions and no longitudinal field (*h*_*i*_=0):ref.^31^


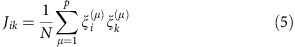


(*cf.* rk *J*_*ik*_=*N* for SK model), where 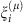 are taken to be independent and identically distributed (i.i.d.) random variables of unit variance. The thermodynamics of this quantum Hopfield model has been worked out in great detail by Nishimori and Nonomura^32^. In fact, that study motivated the development of QA^4^.

When *p* is small (*J*_*ik*_∼1/*N*), it is appropriate to replace local longitudinal fields with their mean values 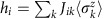. The identity 
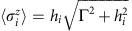
 is used to close this system of equations. For a transverse field below the critical value, Γ<Γ_c_=1, there appears a non-trivial (**m**≠0) solution to the self-consistency equation for the macroscopic order parameter, a vector with *p* components:


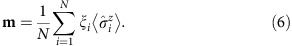


Here, the disorder variables are also written using vector notation: 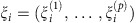.

This model is equivalent to the Curie–Weiss (quantum) ferromagnet, which has a continuous phase transition characterized by a set of mean-field critical exponents. Two of these are particularly useful in the analysis of the annealing complexity: the one for the singular component of the ground state energy 
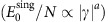
 as well as the dynamical exponent for the gap (*E*_1_−*E*_0_∝*γ*^*b*^), where *γ*=Γ−Γ_c_ is the ‘distance' from the critical point. These exponents are defined for the infinite system, yet fairly general heuristic analysis (see the Methods section) predicts finite-size scaling for the QCP bottleneck:


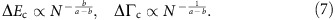


Substituting values *a*=2 and *b*=1/2 for the problem at hand, one may estimate the gap at the critical point and the width of the critical region to be *O* (*N*^−1/3^) and *O* (*N*^−2/3^), respectively.

Worse-than-any-polynomial complexity of quantum annealing might be expected for the first order phase transition, which exhibits no critical scaling (but see ref. 33). Another possibility is for the dynamical exponent to diverge at the infinite randomness QCP: the finite-size gap scales as 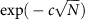 in a random Ising chain^22^. For the Hopfield model, however, this scaling is polynomial, as the disorder is irrelevant at the critical point. More intriguing is the fact that these pessimistic scenarios are not found in SK spin glass either: the model is characterized by the same set of critical exponents, albeit with logarithmic corrections^23^,^24^,^25^. These corrections to scaling increase the gap and, respectively, decrease the width of the critical region by a factor of log^2/9^ *N*. Thus, as long as *T*_ann_≳*N*, non-adiabatic transitions at the critical point should be suppressed. This presents a conundrum as SK model is known to be an NP-hard problem; finding a polynomial-time (even in typical case) quantum algorithm would be a surprising development. The heuristic analysis is clearly insufficient, but ‘digging' deeper into a problem would require a more ‘microscopic' analysis. In the following, the problem is mapped to ordinary quantum mechanics to uncover its low-energy spectrum that explicitly depends on a particular realization of disorder, {***ξ***_*i*_}.

### Exact low-energy spectrum

**Figure 2:**
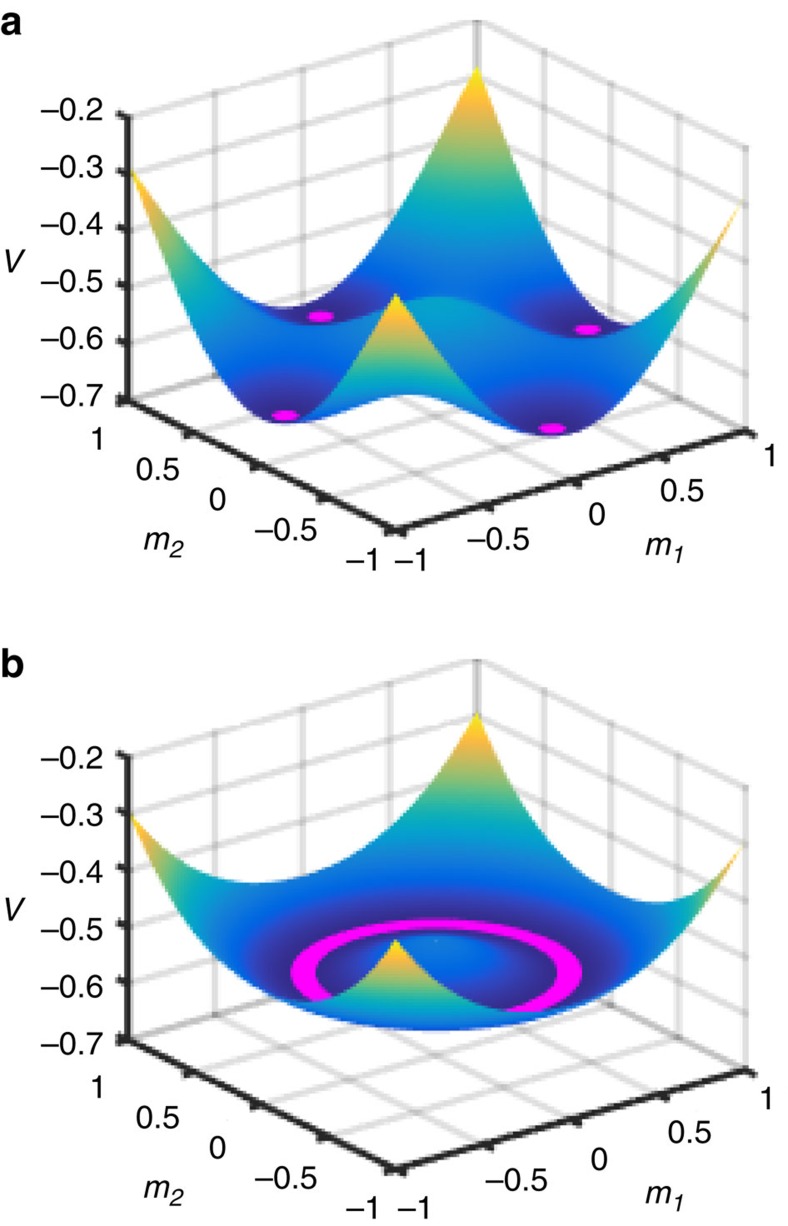
The shape of the effective potential. The plots show the disorder-averaged 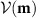 below the critical point (Γ=0.5<Γ_c_) for (**a**) the bimodal distribition and (**b**) the Gaussian distribution of disorder variables. Minima of the potential are highlighted with magenta: the 2*p*-fold degenerate global minima organized in pockets corresponding to encoded patterns in **a**, and a continuum of degenerate minima (connected by arbitrary rotations) in **b**.

Mean-field theory can be derived in a more systematic manner via Hubbard–Stratonovich transformation. General overview is presented below; additional details can be found in Methods and the Supplementary Note 1. Finite-temperature partition function *Z*(*β*)=Tr(*e*^−*βH*^) can be written as a path integral over **m**(*t*), which now acquires a dependence on the imaginary time 0⩽*t*⩽*β*, with periodic boundary conditions: **m**(*β*)=**m**(0). The value of the integral is dominated by stationary paths corresponding to the minimum of an effective potential 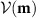. While the discussion above has been deliberately equivocal on the distribution of disorder variables, it is now instructive to contrast bimodal 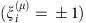 and Gaussian choices. The shapes of the effective potential for both scenarios are depicted in Fig. 2.

The conventional bimodal choice defines the model of associative memory: In the limit Γ=0 the ‘patterns' can be perfectly recalled (*s*_*i*_=±*ξ*^(*μ*)^) when *p* is small. For the Gaussian choice, the global minimum corresponds to a mixture^34^


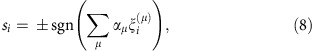


rendering memory useless. In the bimodal case, such ‘spurious' states only become stable once the number of patterns scales with the problem size^35^: *p*>0.05*N*. The BC_*p*_ (bimodal) or O(*p*) (Gaussian) symmetry of the effective potential is only approximate, to leading order in *N*. The degeneracy of the ground state is 2 (due to global spin inversion symmetry) for almost all disorder realizations when *p*⩾3 or *p*⩾2 in the bimodal and Gaussian scenarios, respectively (note that that the *p*=2 bimodal case possesses an additional symmetry, which makes the ground state 4-fold degenerate). The system is in a symmetric superposition of the degenerate global minima at the end of QA. It evolves entirely in the symmetric subspace since the time-dependent Hamiltonian commutes with 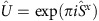. Thus, it should be noted that small gaps between symmetric and antisymmetric wavefunctions are irrelevant to QA and can be ignored.

Disorder fluctuations ‘nudge' QA towards the ‘correct' pattern as it passes the critical point in the bimodal Hopfield model. No further bottlenecks are encountered; gaps for Γ<Γ_c_ as well as the ‘classical' (Γ=0) gap are *O*(1). By contrast, the classical gap scales as *O*(1/*N*) in the Gaussian Hopfield model, alerting to a ‘danger' posed by the Γ=0 ‘fixed point'. To find the low-energy spectrum when Γ<Γ_c_, note that the dominant contribution to the path integral is from paths where the magnitude of the ‘magnetization' vector remains approximately constant, close to its saddle-point value, while the angle is a slow function of time: 
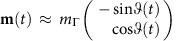
 for *p*=2. Integrating out the amplitude degrees of freedom, the partition function is rewritten as


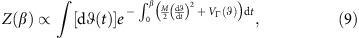


which describes a quantum-mechanical particle of mass *M*=*O*(*N*) moving on a ring, subjected to a random potential


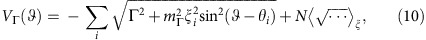


where 
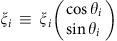
 and the last term, written in shorthand, adds a constant offset so that 〈*V*(*ϑ*)〉_***ξ***_=0. Notice that 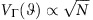 via central limit theorem, thereby representing a higher-order correction to the extensive part of the free energy.

Since the partition function 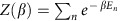 encodes information about the spectrum, low-energy (Goldstone) excitations of the many-body problem are in one-to-one correspondence with the energy levels of a quantum mechanical particle, up to a constant shift. The next step is to find the properties of this potential near a global minimum. These are relevant in a regime away from the critical point, Γ<<Γ_c_, where the universal behaviour characterized by the appearance of ‘hard' bottlenecks sets in.

### Evolution of the random potential

Scaling of the gap for Γ<Γ_c_ can be obtained via semiclassical analysis. Small level splitting due to tunnelling between wells at the two degenerate global minima (this degeneracy is a consequence of the global Z_2_ symmetry: *V*_Γ_(*ϑ*+*π*)=*V*_Γ_(*ϑ*)) is not relevant to QA. Higher degeneracies are statistically unlikely; quantization rules predict *O*(*N*^−1/4^) gaps between energy levels within the symmetric subspace. But this refers to the typical gap, obtained for fixed Γ for almost all realizations of disorder. As quantum annealing sweeps the transverse field for a fixed realization of disorder, *V*_Γ_ (*ϑ*) might undergo global bifurcation. This would result in a small tunnelling gap for a specific value of Γ when the competing minima are in resonance.

**Figure 3:**
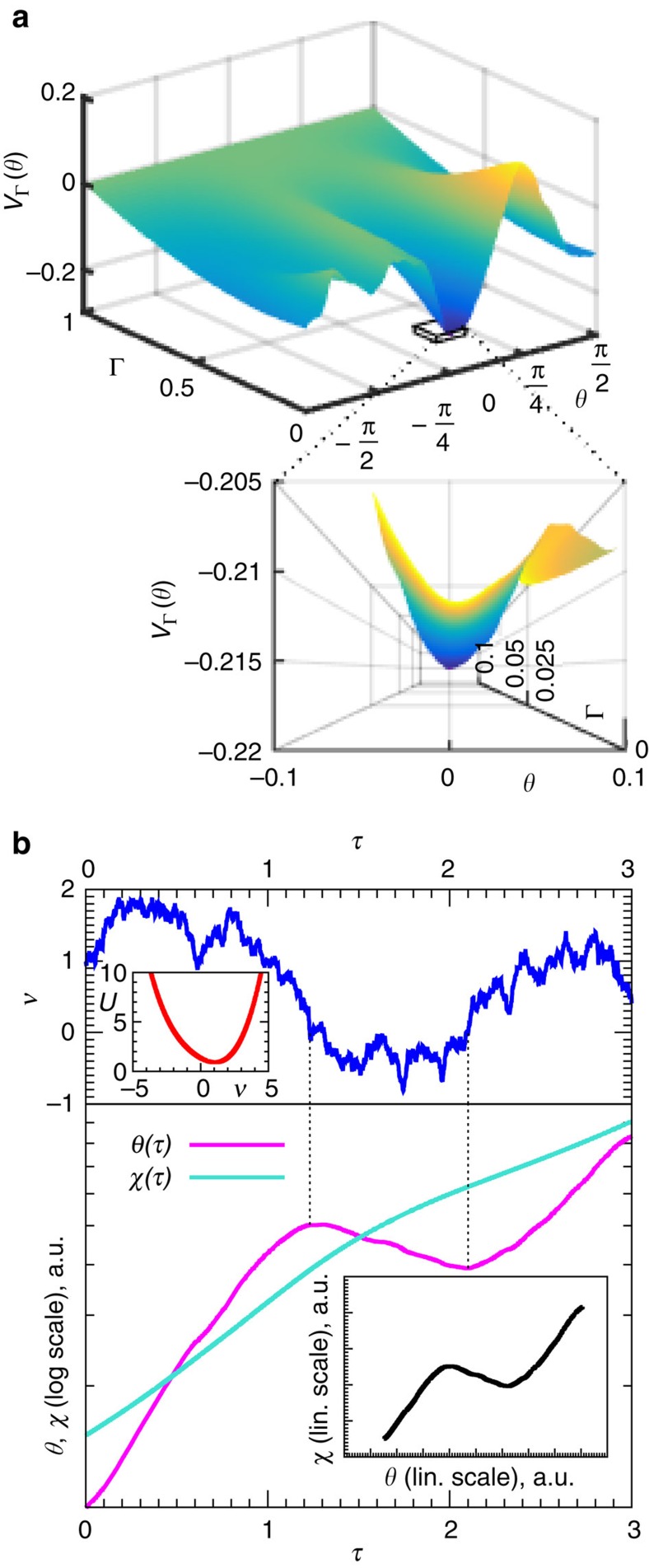
Appearance of local minima. (**a**) Potential *V*_Γ_(*θ*) for a specific realization of disorder as a function of Γ. A perspective-projection three-dimensional plot that zooms on a region near the global minimum of *V*_0_(*θ*) is shown below. (**b**) The top part plots a specific realization of a stochastic process *ν*(*τ*) for the Langevin potential 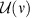 shown in the inset. The bottom part sketches *χ*(*τ*) and *ϑ*(*τ*) (up to a constant factor) obtained by integrating equation (12). The inset is a parametric plot *χ*(*ϑ*) using linear scale. Fluctuations with *ν*(*τ*)<0 (between the dotted vertical lines) are responsible for the appearance of local minima. a.u., arbitrary units.

Such a scenario is impossible near the QCP. Coefficients in the Fourier expansion of the random potential, Σ_*k*_(*a*_*k*_ cos 2*kϑ*+*b*_*k*_ sin 2*kϑ*), decrease as *m*^2*k*^/Γ^2*k*−1^ so that the first harmonic dominates for Γ≈Γ_c_. Semiclassical analysis confirms a *O*(*N*^−1/3^) gap at the critical point (where the curvature of the effective potential vanishes, leaving only the quartic part). As Γ decreases, the random potential becomes more rugged (see Fig. 3a), smooth only on scales Δ*ϑ*∼Γ, which makes global bifurcations more likely. Furthermore, it exhibits properties that allow one to make important predictions without detailed calculations. Rescaling the potential in the vicinity of either global minimum 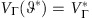,


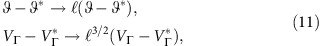


describes the same model but for the rescaled 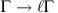 and a different realization of disorder. This scale invariance is responsible for the logarithmic scaling of the number of tunnelling bottlenecks as has been explained earlier in the text. However, it still remains necessary to demonstrate that the density of bottlenecks is non-zero, which entails an examination of the properties of the random potential in the limit Γ=0.

**Figure 4:**
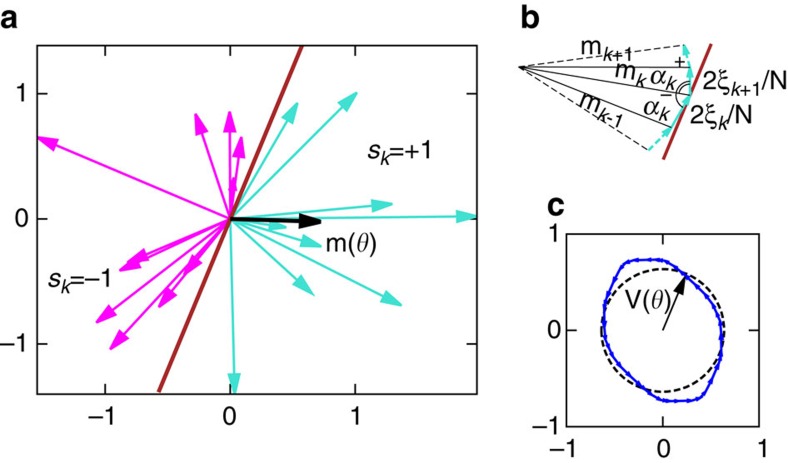
Geometric interpretation of the random potential. Illustration for a random instance with *N*=20. (**a**) A vector **m**(*ϑ*) (black arrow) is defined by drawing a separating line at angle *ϑ* (in brown) and assigning spins with ***ξ***_*k*_ on either side of separating line values *s*_*k*_=+1 (turquoise arrows) and *s*_*k*_=−1 (magenta arrows), respectively. (**b**) Cartoon shows how **m**(*ϑ*) changes as the separating line rotates counterclockwise for increasing *ϑ*: the vector **m**(*ϑ*) is incremented/decremented by (2/*N*)***ξ***_*k*_ (turquoise arrows) whenever *s*_*k*_ changes sign. (**c**) These increments form a closed path, approximated by a circle; fluctuations around the ideal circle (dashed black line) define a random potential (in blue).

The classical optimization problem corresponds to maximizing the magnitude of 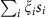. A necessary condition for a local minimum is that two sets of vectors, {***ξ***_*i*_|*s*_*i*_=+1} and {***ξ***_*i*_|*s*_*i*_=−1}, can be separated by a line. As the angle of this line changes, fluctuations of the amplitude give rise to a random potential *V*_0_ (*ϑ*) (see Fig. 4). This suggests a linear-time algorithm for finding a global minimum: Sort all vectors by angle (this may introduce an extra log *N* factor due to sorting overhead) and exhaustively check all possible angles. Of course, QA algorithm is too generic to exploit the specific structure of the problem; moreover *ad hoc* efficient algorithms are unlikely to exist for more general spin-glass problems.

On short intervals, the random process is described as an undamped Langevin process^36^,^37^ in the continuous (*N*→∞) limit (hence the exponent of 3/2 in equation (11), corresponding to its fractal dimension). Properly taking into account the statistics of the extremal properties, the process must be conditioned on the fact that 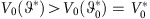 away from the global minimum. As described in Methods and the Supplementary Notes 2 and 3, such a conditioned process consists of two uncorrelated branches, 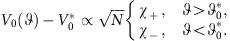 Integrating equations


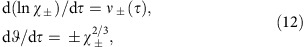


defines *χ*_+_(*ϑ*) and *χ*_−_(*ϑ*) parametrically, in terms of random processes *ν*(*τ*) that correspond to a Brownian motion in a non-linear potential depicted in Fig. 3b. This potential is biased towards positive ‘velocities' ν so that 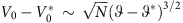 from equation (12). It will, however, experience arbitrary percentage drops due to subpaths with *ν*<0 (albeit with decreasing probability).

**Figure 5:**
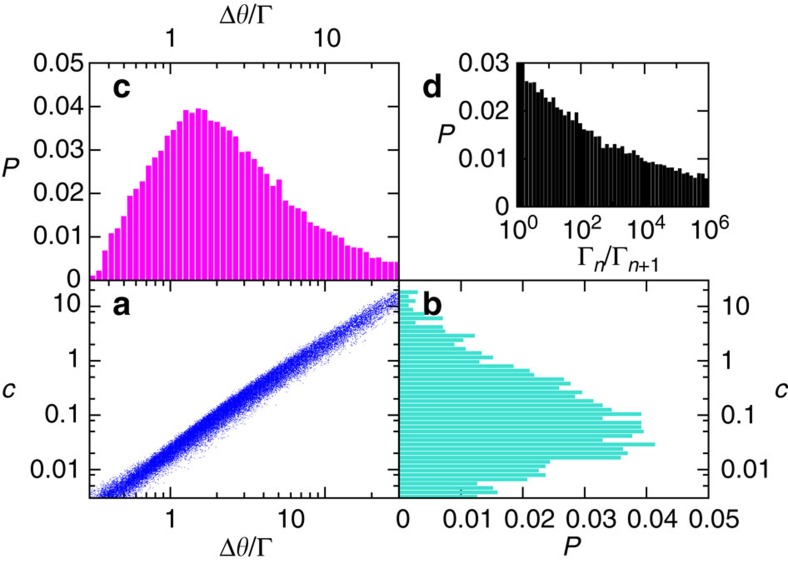
Numerical results. (**a**–**c**) The L-shaped figure formed by the three panels shows the distribution of the tunnelling jumps and the tunnelling exponents. Here Δ*ϑ*/Γ is proportional to the number of spin flips for the tunnelling event, normalized by Γ*N*. The distribution of the tunnelling gaps is quantified using a prefactor in the exponent of equation (4), namely *c*=|ln Δ*E*_tunn_|/(Γ*N*)^3/4^. The panel **a** is a scatterplot of *c* and Δ*ϑ*/Γ. The histograms of univariate distributions of *c* and Δ*ϑ*/Γ (‘projections' of the scatterplot) are plotted in panels **b** and **c**, respectively. (**d**) A histogram of distribution of Γ_*n*_/Γ_*n*+1_ for the successive tunnelling events.

For small but finite Γ, the ‘separating line' becomes blurred. The random potential adds a ‘quantum correction' (see Methods and the Supplementary Note 2), 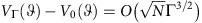. For two minima to come into resonance, they cannot be more than Δ*ϑ*∼Γ apart (that is, *O* (*N*Γ) spin flips). The tunnelling exponent is given by under-the-barrier action 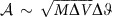, where the effective mass *M*∼*N*/Γ^2^ and 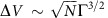, leading to equation (4). Numerical results for the universal distribution of tunnelling jumps and exponents are exhibited in Fig. 5.

## Discussion

Poor scalability of classical annealing of spin-glass models had been linked to the phenomenon of temperature chaos^38^. Interestingly, its existence in mean-field glasses had been debated^39^,^40^,^41^, although it is uncontroversial in finite-dimensional models^42^,^43^. Similarly, failures of quantum annealing might be attributed to transverse field chaos. The phenomenon described in this manuscript represents a much stronger finding. The mere fact that ground states at Γ and Γ+ΔΓ will have vanishingly small overlap as *N*→∞ is not inconsistent with the continuously evolving ground state and poses no ‘threat' to QA. By contrast, ‘hard' bottlenecks correspond to isolated discontinuities that persist as ΔΓ→0.

To dwell upon the generality of these results, first note that scaling of the tunnelling exponent will depend on the universality class of the model. The SK model, for instance, exhibits different scaling of barrier heights, believed to be ∝*N*^1/3^ (see for example, ref. 44). In the model studied, the decrease in the number of spins involved in tunnellings offsets the divergence of the effective mass in the classical limit Γ→0. As the height of the barriers also decreases, the tunnelling gaps widen towards the end of the algorithm. One might expect qualitatively similar behaviour in realistic spin glasses.

The logarithmic scaling of the number of bottlenecks is due to self-similar properties of the free energy landscape in the interval [Γ_min_, Γ_c_]. The lower cutoff should correspond to the smallest energy scale in the classical limit, which for the SK model is also a negative power of *N*, namely 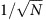. This is related to the linear vanishing of the density of distribution of effective fields as *h*→0 at zero temperature^45^ (since 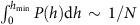). The picture is less clear for CSPs, where the energies are constrained to be non-negative integers; that is, the classical gap is *O*(1). These energy levels have exponential degeneracy, which is lifted by the transverse field. A value sufficient to make the spectrum effectively quasi-continuous might serve as an appropriate lower cutoff Γ_min_ in problems of this type. Lack of ‘hard' bottlenecks in the Hopfield model with the bimodal distribution of disorder and *p*=*O* (1) could be attributed to the fact that the number of valleys is finite, which is not representative of a true spin glass.

An interesting observation is that since ‘hard' bottlenecks correspond to Landau–Zener crossings, annealing times need not be exponentially small. The probability that QA fails to follow the ground state every single time in *n* repeated experiments is


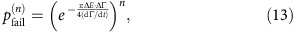


which exactly matches the probability of failure for the annealing rate that is *n* times slower. Using shorter annealing cycles with many repetitions can minimize the effects of decoherence. It suffices that non-adiabatic transitions are suppressed at the critical point only, 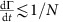.

Even with polynomial annealing rates, coherent evolution would require much better isolation from the environment than what is currently feasible. The only commercial implementation of QA (D-Wave) must contend with a fairly strong coupling to a thermal bath. On the positive side, it accommodates faster annealing cycles, acting as a ‘safety valve' to dissipate any heat generated during the non-adiabatic process. At the same time, it all but ensures that the system is always in thermal equilibrium with the environment.

The theory presented here breaks down when Γ<*T* so that quantum bottlenecks described here may not be a limiting factor if ln (Γ_c_/*T*)≲1/*α*. Main source of errors will be from exponentially many thermally occupied excited states. If the annealing profile were adjusted so that the energy spacing increased beyond *T* towards the end, this would effectively implement classical annealing. An intricate relationship between temperature, problem size, and the properties of the spin-glass model might determine which mechanism (quantum or classical annealing) will be dominant.

Yet another tradeoff in the design of D-Wave chip is a quasi-two-dimensional (2D) topology of interactions *J*_*ik*_ due to fabrication constraints, which incurs significant performance penalty when mean-field models are ‘embedded' into a ‘Chimera' graph^46^. So-called ‘native' problems corresponding to uniformly random instances on this quasi-2D lattice have been argued to be poor candidates for QA due to the lack of a finite-temperature classical phase transition^47^; at the same time, a quantum phase transition at Γ_c_>0 is expected in 2D glasses^48^.

Whereas first-order phase transition immediately implies exponential complexity, even for small sizes, problems having a continuous phase transition may remain tractable up to a threshold, *N*_c_, beyond which tunnelling bottlenecks become dominant (*α* ln *N*_c_∼1). This ‘tractability threshold' serves as a silver lining fot this otherwise negative result. Moreover, the picture of ‘hard' bottlenecks may be equally applicable to classical annealing. A recent study demonstrated that classically ‘hard' instances that exhibit temperature chaos also take much longer time to solve on a D-Wave machine^49^. While in some crafted examples classical annealing is at a unique disadvantage due to first-order phase transition^50^, for most interesting problems both classical and quantum transitions are second-order. In such a scenario, the density of bottlenecks becomes a tie-breaker for evaluating relative performance. Whether quantum annealing can be advantageous in terms of this metric and determining which models will benefit is a practically important question for follow-up work.

There remains a question whether the failure mechanism described here can be somehow circumvented. Such a feat is feasible, for example, for a disordered Ising chain, where an exponentially small gap develops at the critical point, which is a manifestation of Griffiths singularities^22^. Modification of QA that requires controlling the transverse field for each spin individually can suppress these singularities and restore a polynomial gap.

Based on comparison with another exactly solvable model, it seems that frustration—in addition to disorder—is essential for the appearance of small gaps in the spin-glass phase proper. The seemingly random profile of the energy landscape for finite Γ heralds difficulties in avoiding these bottlenecks in generic spin glasses. Although any prospects of exponential speedup should be met with skepticism, heuristics inspired by spin-glass theory revolutionalized branch-and-bound algorithms for CSPs^51^. One can remain hopeful that theoretical advances can similarly aid quantum optimization.

## Methods

### Scaling analysis

Finite-size scaling of the gap at QCP is best understood using an example of a finite-dimensional system. In thermodynamic limit, both correlation length and characteristic time diverge near the phase transition as


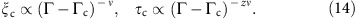


In a finite system this divergence is smoothed out as soon as the correlation length becomes comparable to lattice size (*ξ*_c_∼*N*^1/*d*^). The minimum gap (the reciprocal of *τ*) and the width of the critical region should scale as *N*^−*z*/*d*^ and *N*^−1/(*dν*)^, respectively. In this paper, the product *zν* has been labelled as exponent *b*. Singular behaviour of normalized ground state energy (free energy) is related to the specific heat exponent (*a*=2−*α*). Dimensionality *d* can be eliminated with the aid of hyperscaling relation 2−*α*=(*d*+*z*)*ν* to yield equation (7) of the main text. Independent estimates of the specific heat exponent can be obtained from the exponents for the order parameter and the susceptibility (2−*α*=2*β*+*γ*).

### Mapping to quantum mechanics

Finite-temperature partition function can be written as a sum over a set of paths [*s*_*i*_(*t*)] with 0⩽*t*⩽*β*, where *s*_*i*_ (*t*) alternates between the values ±1. Hubbard–Stratonovich transformation can be used to rewrite it as a path integral


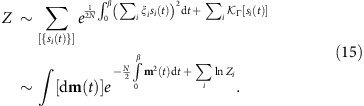


The ‘kinetic' term 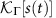 in the first equation penalises kinks, representing Γ-dependent ferromagnetic couplings between Trotter slices. As the interaction term is decoupled, the problem reduces to that of evaluating the single-site partition function *Z*_*i*_ for a spin subjected to a magnetic field with the transverse component Γ and the ‘time-dependent' longitudinal component *h*_*i*_ (*t*)=***ξ***_*i*_**m** (*t*). To leading order in *N*, the saddle-point of the path integral (15) is a solution of mean-field equations. This becomes a degenerate manifold for Gaussian disorder distribution; to determine higher-order contribution all paths such that |*m*(*t*)|=*m*_Γ_ are considered. Evaluating *Z*_*i*_ is best performed in the adiabatic basis that diagonalizes the 2-level Hamiltonian 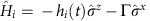,


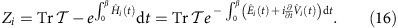


Here 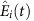 is diagonal with eigenvalues 
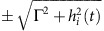
. Its fluctuations around the mean give rise to the random potential *V*_Γ_(*ϑ*). Non-adiabatic terms due to rotation of the basis 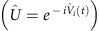 are treated using second order perturbation theory, giving rise to a kinetic term ∝(d*ϑ*/d*t*)^2^. Note a simple form of the perturbative term in equation (16) owing to the fact that 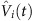 commute for all *t*. The details of this calculation are given in Supplementary Note 1.

### Continuous limit

In the limit *N*→∞, the random potential *V*_Γ_(*ϑ*) defined in equation (10) in the main text converges to a Gaussian process that can be specified completely by its covariance matrix 〈*V*_Γ_(*ϑ*)*V*_Γ′_(*ϑ*′)〉. This can be ‘diagonalized', alternatively expressing the random potential as a linear combination of white-noise processes {*ζ*_*n*_(*ϑ*)}. One representation, as a convolution series 
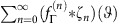
 with kernels


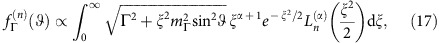


relies on orthogonal properties of associated Laguerre polynomials. The choice *α*=1 ensures that only *n*=0 term survives in the limit Γ=0. The random potential should satisfy a stochastic equation


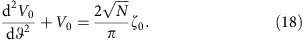


As a side note observe that a similar equation is obtained by taking a continuous limit in the identities that follow from elementary geometry (see Fig. 4 in the main text, 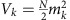):


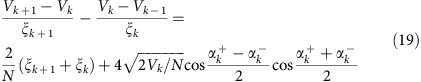


For finite Γ, the form of the potential is modified as follows: It is convolved with a smoothing kernel of width Δ*ϑ*∼Γ, which raises the global minimum by 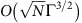. Additional random contributions (from *n*⩾1) have similar scaling. This derivation is presented in greater detail in Supplementary Note 2.

### Extreme value statistics

In the vicinity of the global minimum, the statistics of the classical potential is fundamentally different. The ‘returning force' in equation (18) can be neglected; additionally the process should be conditioned on the fact that it stays above its value at *ϑ*=0 (no generality is lost by choosing the global minimum as the origin). This problem has been a subject of a considerable body of work^36^,^37^, although important aspects ought to be revisited. Here, I present a particularly simple self-contained derivation.

A pair (*χ*,*υ*)—where 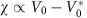 is interpreted as the ‘coordinate' and *υ*=d*χ*/d*ϑ* as the ‘velocity' (*ϑ* being ‘time')—forms a Markov process. The probability distribution *p* (*ϑ*; *χ*, *υ*) satisfies, for *ϑ*>0,


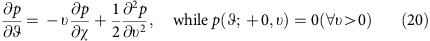


serves as a boundary condition for the absorbing boundary. This probability is ‘renormalized' to condition on the fact that it survives until some Θ≫*ϑ*. It becomes a conserved quantity, but the diffusion equation adds a drift, −∂(log *P*_Θ_)/∂*υ*, repelling from the boundary. The ‘survival' probability *P*_Θ_ in the limit Θ→∞ is, up to ‘time-reversal', the universal asymptotic solution of (20), reduced to ordinary differential equation using scaling ansatz:


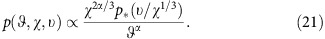


This exploits a fact that fractal dimensions of ‘velocity' and ‘coordinate' are [*υ*]=[*ϑ*]^1/2^ and [*χ*]=[*ϑ*]^3/2^. The asymptotics is dominated by solutions with the smallest possible exponent, *α*=1/4 out of the infinite set of eigenvalues for the ordinary differential equation. This matches a known value obtained with a different method^36^.

The next step performs a change of variables, introducing ‘dimensionless' velocity *ν*=*υ*/*χ*^1/3^, and ‘logarithmic' coordinate *μ*=ln *χ*. With the ‘time' variable redefined via d*τ*=d*ϑ*/*χ*^2/3^, Markov process is described by a tuple (*ϑ*, *μ*, *ν*). Marginalizing out *μ* and *ϑ* in the equation for *p* (*τ*; *ϑ*, *μ*, *ν*) produces Fokker–Planck equation for a stochastic motion of particle in a potential


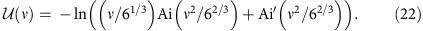


Given a particular realization of *ν*(*τ*), the full process (*μ*, *ν*, *ϑ*) is determined deterministically, by integration (see equation (12) in the main text). The construction of a realization of a random process is performed independently for *ϑ*>0 and *ϑ*<0. More detailed analysis is presented in Supplementary Note 3.

Numerical simulations rescale this random potential instead of evolving the transverse field: 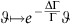 and 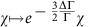. The process is extended to larger values of *τ* as needed (details of the process for small *τ*, where they fall below the numerical precision, are ‘forgotten'). A fairly large range of *τ* is required to gather the sufficient statistics.

### Data availability

The data that support the findings of this study are available from the corresponding author upon request.

## Additional information

**How to cite this article:** Knysh, S. Zero-temperature quantum annealing bottlenecks in the spin-glass phase. *Nat. Commun.* 7:12370 doi: 10.1038/ncomms12370 (2016).

## Supplementary Material

Supplementary Information Supplementary Notes 1-3
