# Supplementary material for: Zero-temperature quantum annealing bottlenecks in the spin-glass phase
Source: Nat Commun. 2016 Aug 5;7:12370. doi: 10.1038/ncomms12370 (PMC4980455; doi:10.1038/ncomms12370)
Supplement: Supplementary Information — Supplementary Notes 1-3 [file ncomms12370-s1.pdf]

Supplementary Information

Supplementary Note 1: Mean-Field Analysis.

The self-consistency equation for the 'magnetisation' vector defined in the main text can be written as follows:

$$\mathbf{m} = \frac{1}{N}\sum_i \boldsymbol{\xi}_i \langle \sigma_i^z \rangle = \frac{1}{N}\sum_i \frac{\boldsymbol{\xi}_i(\boldsymbol{\xi}_i\mathbf{m})}{\sqrt{\Gamma^2 + (\boldsymbol{\xi}_i\mathbf{m})^2}}, \tag{1}$$

where the individual spins polarise along the magnetic field with the components Γ and $h_i = \boldsymbol{\xi}_i\mathbf{m}$ in the transverse and longitudinal directions respectively. Replacing sum over spins with the disorder average, one obtains a non-trivial solution to self-consistency equations for $\Gamma < 1$. For the bimodal distribution, the solutions are $(\pm\sqrt{1-\Gamma^2}, 0, \ldots, 0)$, $(0, \pm\sqrt{1-\Gamma^2}, \ldots, 0)$, *etc.* Other spurious solutions appear for smaller Γ, but they do not become stable for finite p. For Gaussian distribution, the magnetisation vector can have arbitrary direction while the magnitude m_Γ satisfies

$$1 = \int \frac{\xi^2}{\sqrt{\Gamma^2+\xi^2 m_\Gamma^2}} \frac{1}{\sqrt{2\pi}} e^{-\xi^2/2} \mathrm{d}\xi = \frac{1}{\sqrt{2}m_\Gamma} U\Big(\tfrac{1}{2}, 0; \tfrac{\Gamma^2}{2m_\Gamma^2}\Big), \tag{2}$$

where the integral on the right hand side had been converted to that of Mellin type, $\int_0^\infty G_{1,1}^{1,1}\big(\frac{x}{t}\big|\,{}^{1/2}_{0}\big) G_{0,1}^{1,0}\big(t\big|\,{}^{-}_{1}\big)\frac{dt}{t}$ by substituting $t = \xi^2/2$ and $x = \Gamma^2/(2m_\Gamma^2)$. The confluent hypergeometric (Kummer) function on the right hand side can be alternatively expressed in terms of modified Bessel functions.

The low-energy spectrum of the Hopfield model can be obtained by examining the partition function at a finite temperature. It can be written as a sum over paths $[s_i(t)]$ alternating between values $s_i(t) = \pm 1$ with periodic boundary conditions $s_i(\beta) = s_i(0)$:

$$Z \sim \sum_{[\{s_i(t)\}]} e^{\frac{1}{2}\int_0^\beta J_{ik} s_i(t) s_k(t)\mathrm{d}t + \sum_i \mathcal{K}_\Gamma[s_i(t)]}. \tag{3}$$

The 'kinetic' term $\mathcal{K}[s(t)] = (\#\text{ of kinks}) \times \frac{1}{2}\ln\coth\Gamma\Delta t$, where Δt is the discretisation chosen (limit $\Delta t \to 0$ will be taken eventually). This term completely suppresses the dynamics in the limit $\Gamma = 0$.

With $J_{ik} = (1/N)\sum_\mu \xi_i^{(\mu)}\xi_k^{(\mu)}$, two-body interactions are decoupled via Hubbard-Stratonovich transformation: Using the identity $e^{\frac{1}{2N}(\sum_i \boldsymbol{\xi}_i s_i)} \propto \int \mathrm{d}\mathbf{m} e^{-\frac{N}{2}\mathbf{m}^2 + \sum_i \boldsymbol{\xi}_i \mathbf{m} s_i}$, the partition function is recast as a path integral,

$$Z \sim \int [\mathrm{d}\mathbf{m}(t)] e^{-\frac{N}{2}\int_0^\beta \mathbf{m}^2(t)\mathrm{d}t + \sum_i \ln Z_i}, \tag{4}$$

where

$$Z_i = \sum_{[s(t)]} e^{\int_0^\beta h_i(t)s_i(t)\mathrm{d}t + \mathcal{K}_\Gamma[s(t)]} = \mathrm{Tr}\,\mathcal{T} e^{-\int_0^\beta \hat{H}_i(t)\mathrm{d}t} \tag{5}$$

is a single-site partition function for a spin in a 'time-dependent' longitudinal field $h_i(t) = \boldsymbol{\xi}_i\mathbf{m}(t)$. It has been rewritten with the aid of time-ordering operator $\mathcal{T}$. The single-site Hamiltonian $\hat{H}_i(t) = -h_i(t)\hat{\sigma}^z - \Gamma\hat{\sigma}^x$.

The dominant contribution to the path integral is given by the stationary paths, $\mathbf{m}(t) \equiv \mathbf{m}$. To leading order In this case $Z \sim \int \mathrm{d}\mathbf{m} e^{-N\beta\mathcal{V}(\mathbf{m})}$, where the effective potential is

$$\mathcal{V}(\mathbf{m}) = \frac{\mathbf{m}^2}{2} - \Big\langle \sqrt{\Gamma^2 + (\boldsymbol{\xi}\mathbf{m})^2} \Big\rangle_{\boldsymbol{\xi}} = \frac{\mathbf{m}^2}{2} - \sqrt{2}|\mathbf{m}| U\Big(-\tfrac{1}{2}, 0; \tfrac{\Gamma^2}{2\mathbf{m}^2}\Big). \tag{6}$$

The saddle-point value $m_\Gamma = |\mathbf{m}|$ that minimises the potential for $\Gamma = 0$ is given by a solution of eqn. (2).

Considering the higher-order corrections, the dominant contribution comes from the paths where the magnitude of magnetisation fluctuates around the mean-field value and the angle is a slow function of time: $\mathbf{m}(t) \approx m_\Gamma \big({}^{-\sin\vartheta(t)}_{\ \cos\vartheta(t)} \big)$. Since the local field $h_i(t)$ is slow-varying, it is convenient to evaluate the partition function (5) in the adiabatic basis that diagonalises the instantaneous 2-level Hamiltonian: $\hat{H}_i(t) = e^{i\hat{V}_i(t)}\hat{E}_i(t)e^{-i\hat{V}_i(t)}$ where

$$\hat{E}_i(t) = -\sqrt{\Gamma^2 + h_i^2(t)}\,\hat{\sigma}^z \text{ and } \hat{V}_i(t) = \frac{1}{2}\,\mathrm{arccot}\,\frac{h_i(t)}{\Gamma}\hat{\sigma}^y. \tag{7}$$

Using this factorisation, the time-ordered product $\mathrm{Tr}\big(\cdots e^{i\hat{V}_i(t+\Delta t)}e^{-E_i(t+\Delta t)}e^{-i\hat{V}_i(t+\Delta t)}e^{i\hat{V}_i(t)}e^{-E_i(t)}e^{-i\hat{V}_i(t)}\cdots\big)$ can be rewritten in the limit $\Delta t \to 0$ as

$$Z_i = \mathrm{Tr}\,\mathcal{T}e^{-\int_0^\beta (\hat{E}(t) + i\frac{\partial}{\partial t}\hat{V}(t))\mathrm{d}t} \tag{8}$$

The non-adiabatic term can be simply written as $i\frac{\partial}{\partial t}\hat{V}(t)$ since $\hat{V}(t) \propto \hat{\sigma}^y$ commute for all t (should be $-U^\dagger\frac{\partial}{\partial t}U$ more generally); this term acts as a perturbation. The lower energy level, $E_\downarrow(t) = -\sqrt{\Gamma^2 + h_i^2(t)}$, makes a dominant contribution to the partition function in the limit $\beta \to \infty$. Including second-order correction from the perturbation theory, $|\partial V_{\downarrow\uparrow}/\partial t|^2/(E_\downarrow - E_\uparrow)$, one obtains:

$$\ln Z_i \approx \int_0^\beta \bigg(\sqrt{\Gamma^2 + h_i^2} - \frac{\Gamma^2(\partial h_i/\partial t)^2}{8(\Gamma^2 + h_i^2)^{5/2}}\bigg)\mathrm{d}t. \tag{9}$$

As the sum over all sites is performed, the first term in the integrand gives rise to a finite-size correction to the effective potential, which depends on the angle ϑ and a particular realisation of disorder

$$V_\Gamma(\vartheta) = -\sum_i \sqrt{\Gamma^2 + \xi_i^2 m_\Gamma^2 \sin^2(\vartheta - \theta_i)} + N\Big\langle \sqrt{\Gamma^2 + \xi^2 m_\Gamma^2} \Big\rangle_\xi. \tag{10}$$

Vector-valued $\boldsymbol{\xi}_i$ has been parametrised here as $\xi_i\big({}^{\cos\vartheta(t)}_{\sin\vartheta(t)} \big)$. Offsetting average in eqn. (10) above has been made with respect to Gaussian distribution of the projection of $\boldsymbol{\xi}$ onto $\mathbf{m}$ (its value is independent of the direction). The second term in the integrand of eqn. (9) contributes

$$\int_0^\beta \underbrace{\frac{1}{2}\sum_i \frac{\Gamma^2\xi_i^2 m_\Gamma^2 \cos^2(\vartheta - \theta_i)}{4\big(\Gamma^2 + \xi_i^2 m_\Gamma^2 \sin^2(\vartheta - \theta_i)\big)^{5/2}}}_{M} \Big(\frac{\mathrm{d}\vartheta}{\mathrm{d}t}\Big)^2 \mathrm{d}t. \tag{11}$$

The 'effective mass' M can be replaced by its average value

$$M = \frac{N m_\Gamma}{4\sqrt{2}\Gamma^2} U\Big(\tfrac{1}{2}, -1; \tfrac{\Gamma^2}{2m_\Gamma^2}\Big), \tag{12}$$

which approaches $N/\big(4\sqrt{\pi}\Gamma^2\big)$ in the limit $\Gamma \to 0$. The dynamics becomes more classical as the effective mass diverges. Path integral (4) is then rewritten as

$$Z \sim e^{-N\beta\mathcal{V}(m_\Gamma)} \int [\mathrm{d}\vartheta(t)] e^{-\int_0^\beta \big(\frac{M}{2}(\frac{\mathrm{d}\vartheta}{\mathrm{d}t})^2 + V_\Gamma(\vartheta)\big)\mathrm{d}t}. \tag{13}$$

The low-lying energy levels of the many-body problem are thus equivalent to those of a quantum mechanical particle on a ring.

The approximations made above break down for any finite N when Γ is sufficiently small. To avoid this, and to be able to make more quantitative predictions about the behavior of random potential (10), it is necessary to study the continuous limit $N \to \infty$ directly.

Supplementary Note 2: Continuous Potential.

Observe that the central limit theorem can be applied to the entire two-dimensional (in Γ and ϑ) process $V_\Gamma(\vartheta)$. This Gaussian process can be decorrelated by writing it as an infinite function series $V_\Gamma(\vartheta) = \sum_{n,m} \zeta_{nm} f_{nm}(\Gamma, \vartheta)$ involving independent Gaussian variables ζ_{nm}. Basis functions are determined from the covariance $\langle V_\Gamma(\vartheta) V_{\Gamma'}(\vartheta')\rangle$. Since this correlation depends only on the angle difference $\vartheta - \vartheta'$, it is natural to use $f_{nm}(\Gamma, \vartheta) = \tilde{f}_{nm}(\Gamma)\, e^{im\vartheta}$.

Although the best truncated approximation is obtained by using Karhunen-Loève basis, there is no requirement that $\tilde{f}_{nm}(\Gamma)$ be orthogonal. In fact, since covariance involves averaging over distribution $\mathcal{P}(\xi) = \xi\, e^{-\xi^2/2}$, it is quite convenient to use a set of polynomials that are orthogonal with said weight. Using a set of white noise processes

$$\big\langle \zeta_n(\vartheta)\, \zeta_{n'}(\vartheta')\big\rangle = \delta_{nn'}\, \delta(\vartheta - \vartheta') \text{ for } \vartheta, \vartheta' \in [-\pi/2; \pi/2], \tag{14}$$

in lieu of discrete random variables, the series expansion becomes

$$V_\Gamma(\vartheta) = \sqrt{N} \sum_{n=0}^{\infty} \int_{-\pi/2}^{\pi/2} f_\Gamma^{(n)}(\vartheta - \theta)\, \zeta_n(\theta)\, \mathrm{d}\theta, \tag{15}$$

where a factor $\sqrt{N}$ had been introduced for convenience. The convolution kernels should be chosen so as to match the covariance. One suitable choice is

$$f_\Gamma^{(n)}(\vartheta) = A_n \int_0^\infty \sqrt{\Gamma^2 + m_\Gamma^2 \xi^2 \sin^2\vartheta}\, \xi^{\alpha+1} e^{-\xi^2/2} L_n^{(\alpha)}\Big(\tfrac{\xi^2}{2}\Big) \mathrm{d}\xi. \tag{16}$$

Since associated Laguerre polynomials $L_n^{(\alpha)}(t)$ form a complete orthogonal (with respect to weight $t^\alpha e^{-t}$) set of basis functions, it follows that

$$\sum_{n=0}^{\infty} f_\Gamma^{(n)}(\vartheta) L_n^{(\alpha)}\Big(\tfrac{\xi^2}{2}\Big) = \frac{2^\alpha \mathbf{\Gamma}(n+\alpha+1) A_n}{n!\, \xi^\alpha} \sqrt{\Gamma^2 + m_\Gamma^2 \xi^2 \sin^2\vartheta}. \tag{17}$$

Here bold $\mathbf{\Gamma}(x)$ is used to denote the gamma-function to avoid confusion with the transverse field variable. Representing $V_\Gamma(\vartheta)$ using the ansatz of eqn. (15) above one can write the expression for the covariance matrix. Using identities (16) and (17), it is transformed as follows:

$$\begin{aligned}\big\langle V_\Gamma(\vartheta) V_{\Gamma'}(\vartheta')\big\rangle &= \sqrt{N} \sum_{n=0}^{\infty} \int_{-\frac{\pi}{2}}^{\frac{\pi}{2}} f_\Gamma^{(n)}(\vartheta - \theta) \big\langle \zeta_n(\theta) V_{\Gamma'}(\vartheta')\big\rangle \mathrm{d}\theta \\ &= N A_n \sum_{n=0}^{\infty} \int_{-\frac{\pi}{2}}^{\frac{\pi}{2}} f_\Gamma^{(n)}(\vartheta - \theta) \times \\ &\quad \int_0^\infty \sqrt{\Gamma'^2 + m_{\Gamma'}^2 \xi^2 \sin^2(\vartheta' - \theta)}\, \xi^{\alpha+1} e^{-\xi^2/2} L_n^{(\alpha)}\Big(\tfrac{\xi^2}{2}\Big)\, \mathrm{d}\xi\, \mathrm{d}\theta \\ &= \frac{2^\alpha \mathbf{\Gamma}(n+\alpha+1) N A_n^2}{n!} \int_{-\frac{\pi}{2}}^{\frac{\pi}{2}} \int_0^\infty \sqrt{\Gamma^2 + m_\Gamma^2 \xi^2 \sin^2(\vartheta - \theta)} \times \\ &\quad \sqrt{\Gamma'^2 + m_{\Gamma'}^2 \xi^2 \sin^2(\vartheta' - \theta)}\, \xi\, e^{-\xi^2/2} \mathrm{d}\xi\, \mathrm{d}\theta. \end{aligned} \tag{18}$$

This matches the correct value obtained by replacing sum over sites by disorder averages and determines the normalisation constant:

$$A_n = \sqrt{\frac{n!}{2^\alpha \pi \mathbf{\Gamma}(n+\alpha+1)}}\,. \tag{19}$$

Notice that the value of α can be chosen freely: as mentioned above, the decomposition is not unique. The most convenient choice, $\alpha = 1$, leads to the vanishing of all convolution kernels with $n \geqslant 1$ in the limit $\Gamma = 0$. Introducing the rescaled potential via $V_0(\vartheta) = \big(2\sqrt{N}/\pi\big) \chi(\vartheta)$, it is straightforward to verify that

$$\chi(\vartheta) = \int_{-\frac{\pi}{2}}^{\frac{\pi}{2}} \tfrac{1}{2} \big|\sin(\vartheta - \theta)\big|\, \zeta_0(\theta)\, \mathrm{d}\theta \tag{20}$$

satisfies the following stochastic equation:

$$\frac{\mathrm{d}^2\chi}{\mathrm{d}\vartheta^2} + \chi = \zeta_0(\vartheta). \tag{21}$$

The solution is determined uniquely by enforcing periodic boundary conditions: $\chi(-\pi/2) = \chi(\pi/2)$ and $\chi'(-\pi/2) = \chi'(\pi/2)$.

Now considering a finite $\Gamma > 0$, it becomes convenient to reexpress $n = 0$ term in the functional series as a convolution with $\chi(\vartheta)$ instead. This can be accomplished by substituting the left hand side of (21) into eqn. (15) and performing integration by parts. Other terms can also be more conveniently expressed by rewriting white-noise processes in terms of continuous processes: realisations of periodic brownian motion

$$\frac{\mathrm{d}\eta_n}{\mathrm{d}\vartheta} + \bar{\zeta}_n = \zeta_n(\vartheta). \tag{22}$$

Here $\bar{\zeta}_n = \frac{1}{\pi} \int_{-\pi/2}^{\pi/2} \zeta_n(\vartheta)\, \mathrm{d}\vartheta$ is added to ensure periodicity: $\eta_n(-\pi/2) = \eta_n(\pi/2)$.

Now, the random potential is rewritten as

$$V_\Gamma(\vartheta) = \sqrt{N}\, \frac{m_\Gamma}{\sqrt{\pi/2}} \Big((\mathfrak{f}_\Gamma \star \chi)(\vartheta) + \sum_{n=1}^{\infty} \big(\mathfrak{g}_\Gamma^{(n)} \star \eta_n\big)(\vartheta) + \text{const} \Big). \tag{23}$$

The last term is the angle-independent random variable that absorbs $\{\bar{\zeta}_n\}$. Being a constant offset, it cannot affect the physics; and thus will be neglected. Expressions for the kernels $\mathfrak{f}_\Gamma(\vartheta)$ and $\mathfrak{g}_\Gamma^{(n)}$ are obtained by performing integration by parts in variable ϑ:

$$\mathfrak{f}_\Gamma(\vartheta) = \int_0^\infty \frac{\widetilde{\Gamma}^2 (\widetilde{\Gamma}^2 + \xi^2) \xi^2}{2 \big(\widetilde{\Gamma}^2 + \xi^2 \sin^2\vartheta\big)^{3/2}} e^{-\xi^2/2} \mathrm{d}\xi, \tag{24}$$

$$\mathfrak{g}_\Gamma^{(n)}(\vartheta) = \frac{\sin\vartheta \cos\vartheta}{2\sqrt{n+1}} \int_0^\infty \frac{\xi^4 L_n^{(1)}\big(\frac{\xi^2}{2}\big)}{\sqrt{\widetilde{\Gamma}^2 + \xi^2 \sin^2\vartheta}} e^{-\xi^2/2} \mathrm{d}\xi. \tag{25}$$

where the rescaled transverse field $\widetilde{\Gamma} = \Gamma / m_\Gamma$ has been introduced for convenience. Using substitutions $t = \xi^2/2$ and $x = \widetilde{\Gamma}^2 / (2 \sin^2\vartheta)$, the integrals above can be converted to Mellin form and evaluated in terms of special functions:

$$\mathfrak{f}_\Gamma(\vartheta) = \frac{\sqrt{\pi}\, \widetilde{\Gamma}^2}{8 \sin^3\vartheta} \left[3U\Big(\frac{3}{2}, 0; \frac{\widetilde{\Gamma}^2}{2\sin^2\vartheta}\Big) + \widetilde{\Gamma}^2 U\Big(\frac{3}{2}, 1; \frac{\widetilde{\Gamma}^2}{2\sin^2\vartheta}\Big) \right], \tag{26}$$

$$\mathfrak{g}_\Gamma^{(n)}(\vartheta) = \frac{\sqrt{\pi}\, \cos\vartheta}{\sqrt{n+1}\, n!} G_{2,3}^{2,2}\left(\frac{\widetilde{\Gamma}^2}{2\sin^2\vartheta} \,\middle|\, \begin{matrix} 1-n, \frac{1}{2} \\ 1, 2; 0 \end{matrix} \right). \tag{27}$$

Tunneling gaps are associated with global bifurcations of $V_\Gamma(\vartheta)$ which occur mostly in the limit $\Gamma \ll 1$. Moreover, it is this limit that is governed by universal scaling laws and thus will be investigated in greater detail. The kernels above act on scales $\vartheta \sim \Gamma$. For small Γ it is permissible to drop the second term in eqn. (26) and to replace $\sin\vartheta \approx \vartheta$ and $\cos\vartheta \approx 1$ everywhere. It will be seen that any bifurcations also take place for $\vartheta \sim \Gamma$, hence the periodicity of the potential $V_\Gamma(\vartheta)$ becomes irrelevant.

Supplementary Note 3: Extremal Statistics.

The distribution of bottlenecks in the limit $\Gamma \ll 1$ is related to the properties of the classical potential $\chi(\vartheta)$ in a proximity of its global minimum ϑ_0^*. It will be governed by different statistics, derived below, by virtue of this extremality condition.

On short scales, the linear term on the left hand side of eqn. (21) can be neglected in comparison with the large stochastic term $\zeta_0(\vartheta)$. It will be seen self-consistently that the relevant range is $|\vartheta - \vartheta_0^*| = O(\Gamma)$. The stochastic equation describes a free particle in 1D subjected to random force. Specifying the 'coordinate' χ together with the 'velocity' $\upsilon = \mathrm{d}\chi/\mathrm{d}\vartheta$ for some ϑ determines their distribution either in the 'future' ($\vartheta' > \vartheta$) or in the 'past' ($\vartheta' < \vartheta$), where ϑ plays the role of time. This can be described by the following PDE:

$$\frac{\partial p}{\partial \vartheta} + \upsilon \frac{\partial p}{\partial \chi} - \frac{1}{2}\frac{\partial^2 p}{\partial \upsilon^2} = 0, \tag{28}$$

where $p(\vartheta; \chi, \upsilon)$ is the probability density. It will be convenient to perform a shift so that a global minimum coincides with the origin: $\vartheta_0^* = 0$ and $\chi(0) = 0$. Another condition is necessary to ensure that $\chi(\vartheta) > 0$ for $\vartheta \neq 0$. Considering only $\vartheta > 0$ for concreteness, one may introduce an absorbing boundary via boundary condition

$$\lim_{\chi \to +0} p(\vartheta; \chi, \upsilon) = 0 \quad \text{for } \upsilon > 0. \tag{29}$$

Notice that the probability need not vanish as $\chi \to +0$ for negative velocities $\upsilon < 0$. The probability that a random path does not hit this boundary decays as follows:

$$\frac{\partial}{\partial \vartheta} \int p(\vartheta; \chi, \upsilon)\, \mathrm{d}\chi\, \mathrm{d}\upsilon = \int_{-\infty}^{0} \upsilon\, p(\vartheta; +0, \upsilon)\, \mathrm{d}\upsilon. \tag{30}$$

Since the random process is conditioned on the fact that it starts at a global minimum, it is convenient to 'renormalise' the probability so that it becomes a conserved quantity once again. Consider a probability that χ stays positive at least until some distant horizon Θ,

$$q(\vartheta; \chi, \upsilon) \propto p(\vartheta; \chi, \upsilon) \underbrace{\int_{\Theta > 0} P(\Theta; \mathrm{X}, \Upsilon | \vartheta; \chi, \upsilon)\, \mathrm{d}\mathrm{X}\, \mathrm{d}\Upsilon}_{P_\Theta(\chi, \upsilon, \vartheta)}, \tag{31}$$

with an appropriate normalisation factor. The second factor, $P_\Theta(\vartheta; \chi, \upsilon)$ represents the survival probability. It is written as an integral of the Green's function associated with eqn. (28). Time-reversal symmetry implies

$$P(\Theta; \mathrm{X}, \Upsilon | \vartheta; \chi, \upsilon) = P(\vartheta; \chi, -\upsilon | \Theta; \mathrm{X}, -\Upsilon). \tag{32}$$

As a result $P_\Theta(\vartheta; \chi, \upsilon)$ satisfies a PDE obtained from eqn. (28) by changing the sign of $\frac{\partial p}{\partial \vartheta}$ and $\upsilon \frac{\partial p}{\partial \chi}$. With this in mind, the equation for renormalised probability becomes

$$\frac{\partial q}{\partial \vartheta} + \upsilon \frac{\partial q}{\partial \chi} + \frac{\partial}{\partial \upsilon}\left(\frac{1}{P_\Theta}\frac{\partial P_\Theta}{\partial \upsilon} q\right) - \frac{1}{2}\frac{\partial^2 q}{\partial \upsilon^2} = 0. \tag{33}$$

In comparison with eqn. (28), it adds a drift $\propto \partial(\log P_\Theta)/\partial \upsilon$ in addition to the diffusion of 'velocity' υ.

In the limit of large Θ, the 'survival' probability $P_\Theta(\chi, \upsilon, \vartheta)$ corresponds — up to time-reversal ($\upsilon \to -\upsilon$) — to the asymptotic solution of (28),

$$p(\chi, \upsilon, \vartheta) \sim A \frac{p_*(\chi, \upsilon)}{\vartheta^\alpha}. \tag{34}$$

Probability density always converges to this form as $\vartheta \to \infty$, with dependence on the initial conditions only via the prefactor A. Here $p_*(\chi, \upsilon)$ can be thought of as a 'stationary' solution: it satisfies eqn. (28) (with $\partial/\partial\vartheta$ term dropped) and boundary condition (29). Eqn. (28) is invariant with respect to rescaling $(\vartheta, \chi, \upsilon) \mapsto (\ell\vartheta, \ell^{3/2}\chi, \ell^{1/2}\upsilon)$. For this reason, the asymptotic solution should be sought in the form

$$p_*(\chi, \upsilon) = \chi^{2\alpha/3}\, p_*\big(\upsilon/\chi^{1/3}\big). \tag{35}$$

It is possible to simplify equations even further by introducing new 'dimensionless' variables. Defining new 'time' variable via $\mathrm{d}\tau = \mathrm{d}\vartheta/\chi^{2/3}$, it is now a tuple $(\vartheta, \chi, \upsilon)$ that becomes new Markov process. This modification merely adds $\chi^{-2/3}(\partial q/\partial \tau) + \frac{2}{3}(\upsilon/\chi)q$ to the left hand side of eqn. (33). The term $\propto q$ reflects renormalisation of probability density as a result of non-linear transformation.

Furthermore, it is convenient to introduce new 'dimensionless' velocity $\nu = \upsilon/\chi^{1/3}$ and the 'logarithmic' coordinate $\mu = \ln \chi$. The new probability density, appropriately renormalised, reads

$$\bar{q}(\tau; \vartheta, \mu, \nu) = e^{4\mu/3} q\big(\tau; \vartheta, e^{\mu}, \nu\, e^{\mu/3}\big). \tag{36}$$

This is substituted into eqn. (33) which has been modified as described in the previous paragraph. Replacing $\partial/\partial\chi \mapsto e^{-\mu}\big(\partial/\partial\mu - \frac{1}{3}\nu\, \partial/\partial\nu\big)$ and $\partial/\partial\upsilon \mapsto e^{-\mu/3}\partial/\partial\nu$, the following PDE is obtained:

$$\frac{\partial \bar{q}}{\partial \tau} + e^{2\mu/3}\frac{\partial \bar{q}}{\partial \vartheta} + \nu \frac{\partial \bar{q}}{\partial \mu} - \frac{\partial}{\partial \nu}\left(\frac{\partial \mathcal{U}}{\partial \nu}\bar{q}\right) - \frac{1}{2}\frac{\partial^2 \bar{q}}{\partial \nu^2} = 0, \tag{37}$$

where the non-linear potential is written, using eqn. (35), as

$$\mathcal{U}(\nu) \sim \frac{\nu^3}{9} - \ln p_*(-\nu). \tag{38}$$

When $p(\tau; \vartheta, \mu, \nu)$ is marginalised over ϑ, μ, the standard Fokker-Planck equation is obtained. It describes a stochastic process

$$\frac{\mathrm{d}\nu}{\mathrm{d}\tau} = -\mathcal{U}'(\nu)\,\mathrm{sgn}\,\tau + \zeta(\tau), \tag{39}$$

where $\zeta(\tau)$ is the white-noise random force. This stochastic equation can be integrated both forward ($\tau > 0$) and backwards ($\tau < 0$) starting from $\nu(0)$ drawn from the equilibrium distribution $\rho(\nu) \propto e^{-2\mathcal{U}(\nu)}$.

To find the shape of $\mathcal{U}(\nu)$ and $\rho(\nu)$, observe that $p_*\big(\upsilon/\chi^{1/3}\big)$ satisfies a stationary version of eqn. (28), which is reduced to ordinary differential equation. Writing $z = \nu^3/9$,

$$z\psi'' + \frac{2}{3}\psi' + \Big(\frac{2 + 4\alpha}{3} - z\Big)\psi = 0, \tag{40}$$

where $\psi(z) = \sqrt{\rho(z)}$. Disallowing exponentially increasing solutions, $\psi(z)$ is sought in the form $e^{\mp z} f_\pm(z)$ for $z > 0$ and $z < 0$ respectively. This substitution transforms eqn. (40) to hypergeometric form, giving the two branches as $f_+(z) \propto U\left(-\frac{2\alpha}{3}; \frac{2}{3}; 2z\right)$ and $f_-(z) \propto U\left(\frac{2}{3} + \frac{2\alpha}{3}; \frac{2}{3}; -2z\right)$. Matching logarithmic derivatives

$$\frac{\psi'(+0)}{\psi(+0)} = -A\frac{\boldsymbol{\Gamma}\big(\frac{1}{3} - \frac{2\alpha}{3}\big)}{\boldsymbol{\Gamma}\big(-\frac{2\alpha}{3}\big)} \text{ and } \frac{\psi'(-0)}{\psi(-0)} = A\frac{\boldsymbol{\Gamma}\big(1 + \frac{2\alpha}{3}\big)}{\boldsymbol{\Gamma}\big(\frac{2}{3} + \frac{2\alpha}{3}\big)} \tag{41}$$

[where the common factor is $A = 3^{1/3}\boldsymbol{\Gamma}\big(\frac{2}{3}\big)/\boldsymbol{\Gamma}\big(\frac{1}{3}\big)$] requires that

$$\alpha = \frac{1}{4} + \frac{3}{2}n, \tag{42}$$

as verified via Euler's identity $\boldsymbol{\Gamma}(x)\boldsymbol{\Gamma}(1 - x) = \pi/\sin(\pi x)$. Of this infinite set, the smallest positive value $\alpha = 1/4$ has to be chosen. The analytical expression that defines the potential $\mathcal{U}(\nu)$ can be simplified as follows:

$$\psi(\nu) \propto e^{-\mathcal{U}(\nu)} = x\,\mathrm{Ai}(x^2) - \mathrm{Ai}'(x^2), \quad \text{where } x = \nu/\sqrt[3]{6}. \tag{43}$$

Since eqn. (37) is first order in ϑ, μ, these are completely deterministic, given a particular realisation of stochastic process (39):

$$\frac{\mathrm{d}\mu}{\mathrm{d}\tau} = \nu(\tau), \tag{44}$$

$$\frac{\mathrm{d}\vartheta}{\mathrm{d}\tau} = \chi^{2/3}(\tau), \tag{45}$$

with boundary conditions $\chi(-\infty) = \vartheta(-\infty) = 0$. This procedure defines the process $\chi(\vartheta)$ parametrically. So far, a positive branch ($\vartheta > 0$) had been considered. Negative branch ($\vartheta < 0$) is obtained similarly; positive and negative branches are uncorrelated.
